# Intestinal Schwannoma: A Clinicopathological, Immunohistochemical, and Prognostic Study of 9 Cases

**DOI:** 10.1155/2019/3414678

**Published:** 2019-03-26

**Authors:** Zhenbo Shu, Chunsheng Li, Mingze Sun, Zhongmin Li

**Affiliations:** Department of Gastrointestinal, Colorectal, and Anal Surgery, China-Japan Union Hospital of Jilin University, Changchun, China

## Abstract

**Background:**

Intestinal schwannoma is a type of intestinal interstitial tumor with a very low incidence. At present, there are few studies on intestinal schwannoma.

**Methods:**

From January 2010 to January 2018, the patients diagnosed with intestinal schwannoma at the China-Japan Union Hospital of Jilin University were retrospectively reviewed. The patients' clinicopathological features and prognosis were analyzed.

**Results:**

This study enrolled 9 patients with intestinal schwannoma, including 3 males and 6 females. The main symptoms of the patients were abdominal pain and melena. Abdominal computed tomography showed intussusception, slightly high-density shadowing in the intestine, thickening of the intestinal wall, and an intestinal mass. Colonoscopy and endoscopic ultrasonography showed submucosal masses without ulcer formation. Two patients underwent endoscopic biopsy, and the pathological results revealed inflammation and necrosis. One patient had increased neuron-specific enolase (NSE) levels. Immunohistochemical analysis showed that the tumor cells were positive for S-100 and negative for CD117, DOG-1, desmin, and smooth muscle actin. An average of 17 lymph nodes were found around the intestines in 4 patients, all of which demonstrated reactive hyperplasia. No recurrence or metastasis occurred during postoperative follow-up.

**Conclusions:**

Intestinal schwannoma is a rare tumor, and in our study its incidence was higher in women than in men. The main symptoms were abdominal pain and melena. Preoperative increases in NSE levels might contribute to a diagnosis. Complete surgical resection with free negative margins is the standard treatment for benign schwannoma. There was no recurrence or metastasis after complete surgical resection, suggesting that follow-up may not be required.

## 1. Introduction

Schwannomas are tumors originating from Schwann cells that form the neural sheath. They often occur in the peripheral nerves in the extremities, spinal cord, and central nervous system but are rarely seen in the gastrointestinal tract. The incidence of gastrointestinal schwannoma is extremely low, and it mainly occurs in the stomach but rarely occurs in the intestines [1]. At present, there are few studies on intestinal schwannoma, and most of them are case reports [2–5]. Intestinal schwannoma lacks specific clinical manifestations, and its symptoms mainly depend on the size and location of the tumor. The main symptoms include gastrointestinal bleeding, abdominal pain, and changes in bowel habits [6]. Preoperative diagnosis of intestinal schwannoma is very difficult, and it can easily be confused with other mesenchymal tumors, such as a gastrointestinal stromal tumor (GIST) and leiomyosarcoma. Sometimes, intestinal schwannoma must be differentiated from colorectal cancer and melanoma [7, 8]. Colonoscopy, endoscopic ultrasonography (EUS), and abdominal computed tomography (CT) are helpful for diagnosis, but the specificity of these modalities is low. Accurate diagnosis depends on postoperative pathology and immunohistochemistry [8].

Intestinal schwannoma is usually a benign slowly growing tumor and rarely experiences malignant transformation [9]. Surgical resection is the standard method of treatment, and there is a certain probability of local recurrence with incomplete resection. Endoscopic resection is safe and feasible when the tumor is small and grows into the intestine. Open or laparoscopic surgery is mainly used for large tumors that protrude out of the intestines. Laparoscopic surgery has been reported to be advantageous for the treatment of intestinal schwannoma because this technique is minimally invasive and associated with a rapid recovery [5]. There are still controversies regarding the histological grading, surgical indications, and postoperative follow-up for intestinal schwannoma [10, 11]. This study analyzed the clinical manifestations, clinicopathological features, treatment methods, and prognoses of patients with intestinal schwannoma treated in the Department of Gastrointestinal, Colorectal, and Anal Surgery at the China-Japan Union Hospital of Jilin University from January 2010 to January 2018, aiming at providing a basis for the diagnosis and treatment of intestinal schwannoma.

## 2. Patients and Methods

From January 2010 to January 2018, 54 patients diagnosed with schwannoma were admitted to the Department of Gastrointestinal, Colorectal, and Anal Surgery at the China-Japan Union Hospital of Jilin University, including 9 cases involving the intestine, 26 cases involving the stomach, 11 cases involving the retroperitoneal space, 2 cases involving the pelvic cavity, and 6 cases involving other areas, such as the abdominal wall, chest wall, buttocks, and limbs. Nine patients with intestinal schwannoma were numbered from 1 to 9 according to the date of their admission. All patients underwent surgical treatment, and schwannoma was confirmed by postoperative pathology and immunohistochemistry. All patients were followed up once a year after surgery, and none were lost to follow-up. The follow-up time ranged from 10.4 months to 51.4 months, with an average time of 28.4 months.

The basic information collected from the patients included gender, age, tumor location (small intestine, colon, or rectum), and distance from the lower margin of the tumor to the anus. Preoperative laboratory indicators included hemoglobin (male: 120-160 g/L, female: 110-150 g/L), albumin (35.0-52.0 g/L), carcinoembryonic antigen (CEA) (<5.00 ng/mL), carbohydrate antigen 19-9 (CA 19-9) (<37.00 U/mL), and neuron-specific enolase (NSE) (<25.00 ng/mL). The main preoperative auxiliary examinations included colonoscopy and pathological biopsy, EUS, and abdominal CT. The surgical and postoperative pathology information included the surgical procedure, operative time, tumor diameter, cell type of the tumor, depth of invasion, number of detected lymph nodes, and mitotic activity. The immunohistochemical markers included S-100, CD117, DOG-1, CD34, Ki-67, desmin, and smooth muscle actin (SMA). Prognostic indicators included postoperative complications, length of hospital stay, recurrence, and metastasis.

## 3. Results

Among the 9 patients with intestinal schwannoma, there were 3 males and 6 females. The patients' ages ranged from 38 years to 84 years, with an average age of 60.9 years. There were 2 cases involving the small intestine, 1 involving the cecum, 2 involving the ascending colon, 1 involving the descending colon, and 3 involving the rectum. In the 3 cases of rectal schwannoma, the distance from the lower margin of the tumor to the anus was 6 cm, 10 cm, and 10 cm. The main symptoms that patients presented with were abdominal pain (4 patients) and melena (3 patients). Two patients had no obvious symptoms, and the tumor was found on colonoscopy during routine screening examination (Table 1).

**Figure 1:**
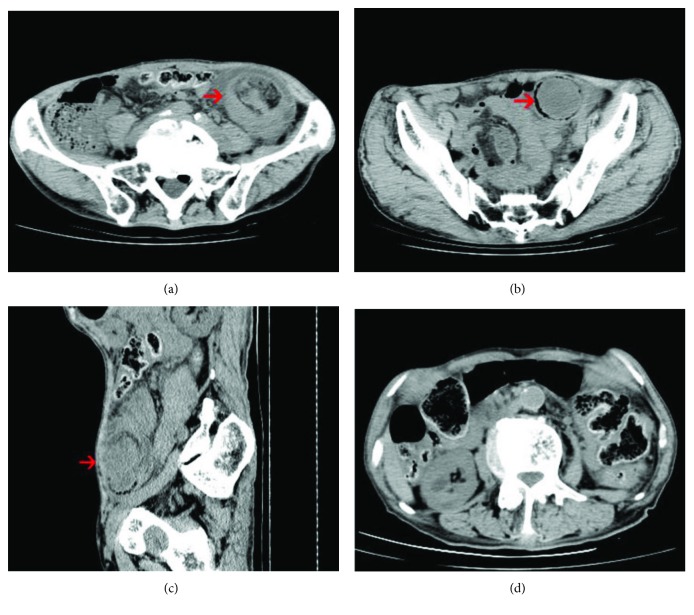
Abdominal CT of Case 1 showing (a) a target sign in the intestinal canal (arrow), (b) colonic schwannoma (arrow), (c) descending colon intussusception (arrow), and (d) intestinal obstruction caused by the schwannoma.

**Figure 2:**
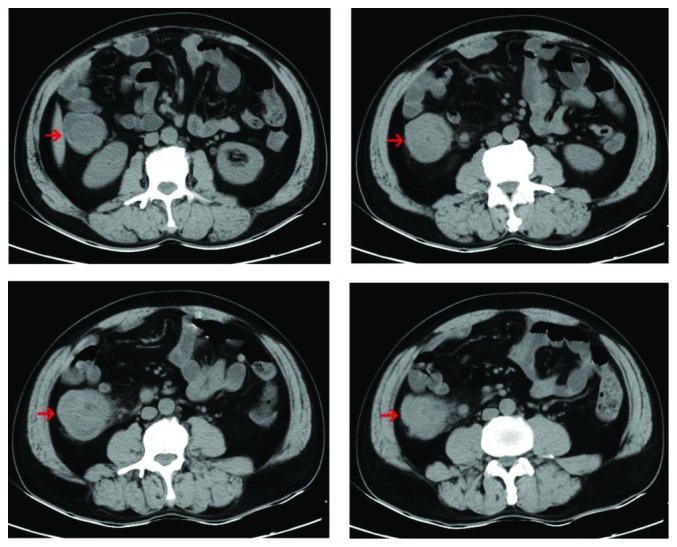
Abdominal CT of Case 9 showing an ascending colon mass and surrounding lymph node enlargement, which was suggestive of the possibility of colon cancer.

**Figure 3:**
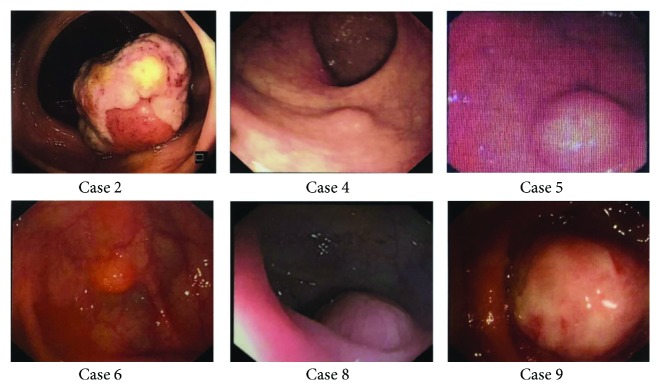
Colonoscopy showing submucosal masses with no obvious ulcers.

**Figure 4:**
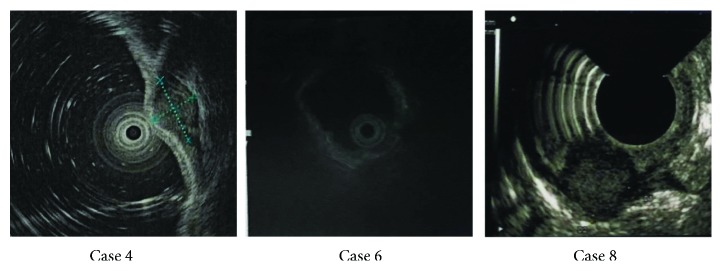
EUS showing submucosal masses mainly located in the submucosa and muscularis propria.

Six patients underwent abdominal CT examinations before surgery, and the other patients did not have CT scan done because of the small size of the tumor. Abdominal CT showed intussusception (Figure 1), low-density shadowing in the intestine, thickening of the intestinal wall, and an intestinal mass. The abdominal CT in Case 9 showed a tumor in the ascending colon with enlarged peripheral lymph nodes, which were suggestive of colon cancer (Figure 2). All patients underwent colonoscopy, except for one patient with intussusception. Colonoscopy mainly showed submucosal protruding masses, and no ulcers were observed (Figure 3). No obvious abnormalities were found in 2 patients with small intestinal schwannomas. The pathological results of biopsies from two patients with suspected colon cancer on CT revealed inflammatory granulation tissue and inflammatory exudate in one patient and necrotic tissue and inflammatory exudate in the other. Four patients underwent EUS, and the results showed submucosal tumor (probably a GIST), muscularis propria lesion (high possibility of a GIST or leiomyoma), submucosal lesion (probably a neuroendocrine tumor), and muscularis propria lesion (high possibility of a GIST) (Figure 4). Two patients had significant anemia, with hemoglobin levels of 68 g/L and 62 g/L. Their albumin levels also decreased (34.2 g/L and 18.9 g/L). Preoperative CEA and CA 19-9 levels were within the normal range. The serum NSE levels in two patients were 17.69 ng/mL and 27.42 ng/mL, the latter of which was higher than the normal value. Four patients were diagnosed with GISTs before surgery, 2 with colon cancer, 1 with a neuroendocrine tumor, 1 with an intussusception, and 1 with gastrointestinal bleeding (Table 2).

All the patients received surgical treatment: 2 patients underwent laparoscopic partial rectal resection, 2 patients underwent right hemicolectomy, 2 patients underwent small intestinal tumor resection, 1 patient underwent partial descending colon resection, 1 patient underwent transanal rectal tumor resection, and 1 patient underwent endoscopic submucosal dissection. The average operative time was 118 minutes. The minimum diameter and maximum diameter of the tumors were 0.8 cm and 5.0 cm, respectively, and the average diameter was 2.4 cm. Postoperative pathology showed that 9 schwannomas were mainly composed of spindle cells, and the tumors were mainly located in the submucosal layer and muscularis propria layer of the intestines, without invading the serosa. An average of 17 lymph nodes were found around the intestines in 4 patients, all of which demonstrated reactive hyperplasia. The mitotic count of two patients was less than 1 per 10 high-power fields (HPF) (Table 3). Immunohistochemical analysis showed that the tumor cells were positive for S-100 and negative for CD117, DOG-1, desmin, and SMA. Cells were positive for CD34 in one case. According to the results of immunohistochemistry, 9 patients were diagnosed with schwannomas. The Ki-67 proliferative indexes (MIB-1) were no more than 3%. Two patients developed incomplete intestinal obstruction and ascites, respectively, and both improved after symptomatic treatment. The average length of hospitalization was 15 days, and no recurrence or metastasis occurred during postoperative follow-up (Table 4).

## 4. Discussion

Intestinal schwannoma arises from the Schwann cells of the neural plexus of the intestinal wall [12] and accounts for approximately 2% to 6% of all submucosal tumors of the intestine. The intestinal neural plexus includes the submucosal neural plexus (Meissner's plexus) and the intestinal myenteric neural plexus (Auerbach's plexus), and schwannomas mainly originate from Auerbach's plexus [13]. Schwannomas were first reported by Verocay in 1910, and Stout described their characteristics in detail in 1935 [14]. In 1988, Daimaru et al. first proposed the concept of gastrointestinal benign schwannoma and fully documented its morphological and phenotypic characteristics [13]. In recent years, with the popularization of colonoscopy screening and improvements in immunohistochemistry technology, an increasing number of cases of intestinal schwannoma have been reported. However, related studies are mainly case reports and reviews, and large sample studies and prospective studies are lacking [2, 15, 16]. Because the incidence of intestinal schwannomas is extremely low, the exact incidence is still unclear and has only been estimated in some existing studies. The incidence of GIST is significantly higher than that of gastrointestinal schwannoma, and the incidence ratio between these two tumors is approximately 50-100 : 1 [17]. Hou et al. reported that benign schwannomas accounted for 2.9% of gastrointestinal mesenchymal tumors [1], while another study showed that 20 of 600 colorectal mesenchymal tumors (3.3%) were schwannomas [8]. According to the World Health Organization's (WHO) classification for tumors of the digestive system, intestinal schwannoma belongs to the class of gastrointestinal mesenchymal tumors (GIMTs) [18]. Some authors have classified intestinal schwannoma as a category of gastrointestinal autonomic neurogenic tumors (GANTs) [19, 20]. However, GANTs are usually composed of spindle cells or epithelial cells and lack the structural features and lymphatic infiltration of schwannomas. Most GANTs express CD34 and CD117, and only approximately one-third express S-100. In addition, the recurrence and metastasis rates of GANTs are as high as 58% [7, 21]. Therefore, there is a significant difference between intestinal schwannomas and GANTs.

Previous studies have suggested that the incidence of intestinal schwannomas was roughly the same in men and women. Intestinal schwannomas can occur in patients of all ages but mainly in people older than 60, with an average patient age of 60-65 years [8, 13]. The average age of the 9 patients in this study was 60.9 years, which was consistent with that in the previous reports. In this study, however, the number of female patients was significantly higher than the number of male patients (6 patients and 3 patients, respectively). A retrospective study in Japan showed that the ratio of males to females in patients with intestinal schwannomas was 21 : 25 [2]. Bohlok et al. reviewed 96 patients with colorectal schwannoma and found that the proportion of women (58.3%) was higher than that of men (41.7%) [6]. Therefore, we speculated that intestinal schwannoma may be more common in women. Most patients with intestinal schwannomas have no obvious symptoms, and tumors are incidentally discovered on colonoscopy. The common symptoms include abdominal pain, melena, hematochezia, difficulty defecating, and tenesmus [6]. The symptoms may be related to the location and size of the tumor, and intussusception may occur when the tumor grows to a certain size [22]. The main symptoms of the patients in this study were abdominal pain and melena, and the average tumor diameter was 2.4 cm. Two patients with small intestinal schwannomas had melena, and no obvious areas of hemorrhage were found on gastroscopy and colonoscopy. Abdominal CT revealed suspicious small intestinal tumors, which were confirmed to be schwannomas during surgery. One patient developed an intestinal intussusception and intestinal obstruction due to a colonic schwannoma and presented with symptoms of abdominal pain, melena, difficulty defecating, and changes in bowel habits. Intussusception caused by schwannoma is very rare in adults [23, 24]. The most common sites of gastrointestinal schwannomas are the stomach and small intestine, accounting for 83% and 12% of the total cases, respectively, while colorectal schwannomas are rare [25]. Schwannomas can occur anywhere in the intestines, and a study from the United States showed that the most common site of intestinal schwannomas was the cecum [8]. However, a retrospective analysis from Japan found that intestinal schwannomas mainly occurred in the rectum (45.6%) [2]. A recent study by Bohlok et al. concluded that schwannomas most commonly occurred in the ascending colon and cecum, followed by the sigmoid colon, rectum, descending colon, and transverse colon [6]. In this study, 3 cases involved the rectum, 2 involved the ascending colon, 2 involved the small intestine, and 1 involved the cecum. We speculate that the differences in the location of schwannomas in this study may have been limited by the number of cases and may also have been affected by other factors, such as race and environment. Therefore, the distribution of intestinal schwannomas needs to be confirmed in large sample studies.

There were no specific changes in routine blood test and liver function test results in patients with intestinal schwannoma, and decreases in hemoglobin and albumin levels might occur when tumors caused hemorrhage. In this study, 2 patients with melena had significant decreases in hemoglobin and albumin levels. The CEA and CA 19-9 levels of the studied patients were within the normal range, which was consistent with the findings of previous studies [26, 27]. Notably, one patient had elevated serum NSE levels. NSE is an acid protease specific to neurons and neuroendocrine cells. It is highly concentrated in nerve cells, neuroendocrine cells, and tumor cells. Immunohistochemistry of gastrointestinal schwannomas shows that NSE results are often positive [28, 29]. However, there are no relevant reports on increases in serum NSE levels in patients with schwannomas of the gastrointestinal tract, which may be related to the lower incidence of schwannoma and the lower detection rate of NSE. In this study, 1 out of 2 patients who had serum NSE levels measured showed increased levels, suggesting that serum NSE may be a potential tumor marker for intestinal schwannoma.

Preoperative diagnosis, though limited due to lack of the sensitivity and specificity of the biopsy or fine needle aspiration (FNA), if obtained can guide to the local resection instead of the radical surgery. Common examinations for intestinal schwannoma include colonoscopy, EUS, abdominal CT, and magnetic resonance imaging (MRI). Colonoscopy often shows a submucosal mass that protrudes into the intestinal lumen, and the surface mucosa is smooth. In a few patients, ulcers may occur and the mucosa may be invaded [30, 31]. As the tumor is hard and located beneath the mucosa, it is difficult to obtain tumor tissue for accurate diagnosis by routine mucosal biopsy taken at endoscopy. In this study, two patients with colonic schwannomas underwent colonoscopic biopsy, which revealed inflamed and necrotic tissue. EUS is also a common examination, and schwannoma mainly presents as a submucosal hypoechoic mass. EUS can determine the location and boundaries of a tumor in the intestinal wall, but it cannot distinguish schwannomas from other mesenchymal tumors [6]. In this study, 4 patients underwent EUS and the tumors were considered GISTs, leiomyomas, or neuroendocrine tumors, indicating that the value of EUS for the diagnosis of schwannoma was relatively low. At present, some studies have attempted to identify submucosal tumors by EUS-guided fine needle aspiration or biopsy (EUS-FNA) combined with immunohistochemistry [32]. EUS-FNA combined with immunohistochemistry can significantly improve the accuracy of the preoperative diagnosis of GISTs, but its application value for schwannomas needs further study. Abdominal CT is a common and effective examination. In abdominal CT, schwannomas are mainly characterized by submucosal homogeneous masses with low-density shadowing on plain scans and mild to moderate homogeneous enhancement on enhanced scans with significantly enhanced enlarged lymph nodes around the tumor. There is no evidence of a tumor capsule, cystic changes, cavity formation, necrosis, or calcifications. These characteristics can help to differentiate schwannomas from GISTs and other malignant tumors [33]. Although enlarged lymph nodes often appear around schwannomas, they are not caused by tumor metastasis. Hou et al. speculated that lymph node enlargement was due to the release of cytotoxin by tumor cells, which induces chemokinesis of lymphocytes [1]. However, Atmatzidis et al. believed that lymph node enlargement was caused by an inflammatory reaction [34]. In this study, one patient with a colonic schwannoma had enlarged lymph nodes around the tumor on an abdominal CT scan. Postoperative pathology revealed an average of 17 lymph nodes around the intestines in 4 patients, all of which demonstrated reactive hyperplasia. Therefore, lymph node enlargement may be associated with inflammation. Abdominal MRI can show the location of the tumor and its relationship with surrounding tissue. Schwannoma has a low signal and moderate to high signal on T1- and T2-weighted images, respectively [35]. However, MRI has a limited role in the identification of schwannoma. Positron emission tomography/CT (PET/CT) is mainly used to distinguish benign tumors from malignant tumors and to detect distant metastasis. PET/CT examinations were used in a few patients with schwannoma. Because schwannomas mainly exhibit hypermetabolic activity, it is difficult to distinguish them from malignant tumors [6, 36, 37]. PET/CT is not currently recommended for routine examination of intestinal schwannoma.

The preoperative diagnosis rate of intestinal schwannoma is relatively low. A study by Inagawa et al. showed that the preoperative diagnostic rate was only 15.2% [2]. The diagnosis depends mainly on postoperative pathology and immunohistochemistry. Microscopically, schwannomas are encapsulated neoplasms composed of elongated bipolar spindle cells. A typical feature is a prominent lymphoid cuff with or without germinal center formation surrounding the tumor cells. Nuclear atypia with hyperchromasia is common, but the mitotic count rarely exceeds 5/50 HPF. Immunohistochemically, intestinal schwannomas are strongly positive for S-100 and are often positive for vimentin and glial fibrillary acidic protein (GFAP) [1, 6]. However, they are typically negative for CD117 (KIT), SMA, desmin, and actin and are usually negative for CD34 [8]. Immunohistochemistry can help to distinguish schwannomas from other GIMTs, such as GISTs, leiomyomas, and leiomyosarcomas. Approximately 98% of intestinal schwannomas are benign, and malignant schwannomas are rarely reported [6, 9, 38, 39]. However, malignant schwannomas were mainly reported before 2000 when the understanding of schwannomas was still limited. Therefore, the malignancy of these tumors may need to be reassessed. There is no clear standard of malignancy for the biological behavior of intestinal schwannoma [30]. Current indicators for reference include the Ki-67 proliferative index (MIB-1), nuclear atypia, mitotic activity rate, and tumor size. For example, malignancy grades of GISTs are based on the tumor size and mitotic activity rate [40]. The Ki-67 proliferative index is an indicator of malignancy. A Ki-67 proliferative index greater than 5% is associated with high invasive potential of the tumor, while a Ki-67 proliferative index greater than 10% is indicative of malignancy [30]. A mitotic activity rate > 5/HPF and a tumor size > 5 cm are associated with a high risk of metastasis and recurrence [41]. A low mitotic activity rate and the absence of nuclear atypia are often the characteristics of benign tumors [30]. The histological grading criteria for intestinal schwannoma need further study. In this study, the Ki-67 proliferative indexes were ≤3% and tumor diameters were ≤5 cm. Moreover, no recurrence or metastasis was observed during follow-up, so all these tumors were considered to be benign schwannomas.

At present, the best treatment for intestinal schwannoma is surgery. For benign schwannomas, complete surgical resection with free negative margins is the standard treatment. Radical surgery and extended resection are unnecessary. The surgical method depends on the location and size of the tumor, and a minimally invasive procedure is recommended. When the tumor is small, endoscopic resection can be performed, and when the tumor is closer to the anus, transanal tumor resection may be considered. Open or laparoscopic partial bowel resection may be performed when the tumor is large and located in the small intestine or colon. Radical surgery is recommended for malignant schwannomas, and the value of chemotherapy and radiotherapy is unclear [6]. In this study, 2 patients with rectal schwannomas underwent laparoscopic partial rectal resection, and they recovered smoothly after surgery. There were no complications after surgery, and the hospitalization time was relatively short. Therefore, laparoscopic surgery can be recommended for the treatment of intestinal schwannoma. Although surgical treatment was performed for all of the discovered intestinal schwannomas in the current reports, the indications for surgery are still unclear. Intestinal schwannomas are benign tumors that grow slowly and have an extremely low rate of malignant transformation. Especially for asymptomatic patients, whether the tumor should be removed once it is discovered is still debatable. The diameter of schwannomas is usually less than 2 cm, and most of these patients have no obvious symptoms. Tumor diameter may be related to clinical symptoms, depth of invasion, and lymph node involvement. In this study, four patients with tumor diameters > 2 cm developed abdominal pain or melena. The EUS showed that most of the tumors in these patients invaded the subserosal layer, the invasion depth of which was significantly deeper than that of tumors less than 2 cm in diameter. In addition, lymph node enlargement was observed in 3 patients with tumor diameters > 2 cm. It can be concluded that the schwannomas with diameters < 2 cm have better biological behavior. A GIST is a borderline tumor with a relatively high risk of malignant transformation. According to the National Comprehensive Cancer Network (NCCN) guidelines, for GISTs with diameters < 2 cm, there is no need for surgical excision if there are no high-risk EUS features (such as irregular borders, cystic spaces, ulcers, ulceration, echogenic foci, and heterogeneity), but periodic endoscopic or radiographic surveillance should be considered [40]. The risk of malignant transformation of intestinal schwannoma is significantly lower than that of a GIST. However, whether schwannomas with diameters < 2 cm can be observed regularly like GIST or should be resected immediately requires more postoperative follow-up and further studies. In addition, it is controversial whether follow-up is necessary after resection of intestinal schwannomas. Kanneganti et al. believed that because schwannomas lack specific histological grading criteria, all patients need regular follow-up [10]. However, Kawaguchi et al. believed that there was no need for follow-up after complete resection [11]. We carefully studied the previous reports and found that some studies determined that there was a risk of recurrence after complete resection of benign schwannomas because they confused schwannomas with GISTs, GANTs, and malignant peripheral schwannomas [19, 20, 42]. Moreover, some studies referred to data regarding soft-tissue neurilemmomas, but there are differences in the origin, tissue composition, and pathological features of gastrointestinal schwannomas and soft-tissue schwannomas [43], so their biological behavior is also inconsistent. At present, no recurrence or metastasis has been reported in patients with benign intestinal schwannomas after complete surgical resection. Similarly, no recurrence or metastasis occurred during the postoperative follow-up in this study. Thus, for the benign intestinal schwannomas that have been completely resected, follow-up may not be necessary.

Some limitations of this study should be addressed. Firstly, this study was limited by its retrospective nature. Secondly, because the incidence of intestinal schwannoma is relatively low, the number of cases in this study was limited. Thirdly, the clinical data of some patients were not comprehensive enough, such as data regarding serum NSE levels and mitotic counts. Finally, the criteria for histological grading of intestinal schwannomas, the surgical indications for intestinal schwannomas, and whether regular follow-up is needed remain unclear and need further study.

## 5. Conclusion

The incidence rate of intestinal schwannoma is very low and significantly lower than that of GISTs. The incidence is slightly higher in women than in men and is highest among people older than 60 years of age. Intestinal schwannomas are benign tumors that rarely exhibit malignant transformation. The preoperative diagnosis rate of intestinal schwannomas is low, and contrast-enhanced abdominal CT examination and EUS-FNA biopsy combined with immunohistochemistry are recommended. Preoperative elevated serum NSE levels may contribute to the diagnosis of schwannoma. Complete surgical resection is the standard treatment for intestinal schwannoma, and laparoscopic resection is safe and feasible. There is no definitive histological grading standard for intestinal schwannoma, and further studies are needed to determine whether asymptomatic or small-diameter intestinal schwannomas should be removed. Postoperative follow-up is not necessary for completely resected and pathologically confirmed benign schwannomas.

## Figures and Tables

**Table 1:** Clinical data of patients with intestinal schwannoma.

Cases	Age	Gender	Tumor location	Main symptoms	Distance from the anus (cm)
1	84	Male	Descending colon	Abdominal pain	NA
2	75	Female	Ascending colon	Abdominal pain	70
3	52	Female	Small intestine	Melena	NA
4	65	Female	Rectum	Asymptomatic	6
5	55	Female	Rectum	Asymptomatic	10
6	38	Male	Cecum	Abdominal pain	NA
7	71	Female	Small intestine	Melena	NA
8	53	Female	Rectum	Melena	10
9	55	Male	Ascending colon	Abdominal pain	65

NA: not available.

**Table 2:** Preoperative examination findings of patients with intestinal schwannoma.

Cases	CT	Colonoscopy	Biopsy	EUS	HGB	ALB	CEA	CA 19-9	NSE	Diagnosis
1	Intussusception	NA	NA	NA	113	36.0	NA	NA	NA	Intussusception
2	Low-density shadow	Protruding mass	Inflammation	NA	68	34.2	NA	NA	NA	Colon cancer
3	Small intestinal mass	Negative	NA	NA	139	45.7	NA	NA	NA	GIST
4	Negative	Submucosal mass	NA	GIST	136	43.2	NA	NA	NA	GIST
5	NA	Submucosal mass	NA	GIST/leiomyoma	110	41.4	0.80	1.46	NA	GIST
6	NA	Submucosal mass	NA	NET	157	46.7	4.00	NA	NA	NET
7	Bowel wall thickening	Negative	NA	NA	62	18.9	NA	NA	NA	GI bleeding
8	NA	Submucosal mass	NA	GIST	138	41.1	0.89	5.26	17.69	GIST
9	Ascending colonic mass	Protruding mass	Inflammation	NA	125	39.2	0.38	6.36	27.42	Colon cancer

EUS: endoscopic ultrasonography; NA: not available; GIST: gastrointestinal stromal tumor; NET: neuroendocrine tumor; GI: gastrointestinal.

**Table 3:** Surgical and pathological features of patients with intestinal schwannoma.

Cases	Surgery	Operative time	Diameter	Cell type	Invasion depth	Lymph node	Mitotic count
1	Partial descending colon resection	160	4.0	Spindle cell	Submucosa-subserosa	18	0-1/10 HPF
2	Right hemicolectomy	120	4.0	Spindle cell	Muscularis propria-subserosa	17	0-1/10 HPF
3	Small intestinal tumor resection	135	4.0	Spindle cell	Submucosa-subserosa	NA	NA
4	Transanal rectal tumor resection	35	0.8	Spindle cell	Submucosa-muscularis propria	NA	NA
5	Laparoscopic partial rectal resection	160	1.8	Spindle cell	Submucosa-subserosa	NA	NA
6	Endoscopic submucosal dissection	40	0.6	Spindle cell	Submucosa	NA	NA
7	Small intestinal tumor resection	140	0.8	Spindle cell	Submucosa-muscularis propria	NA	NA
8	Laparoscopic partial rectal resection	120	1.0	Spindle cell	Submucosa-muscularis propria	7	NA
9	Right hemicolectomy	150	5.0	Spindle cell	Muscularis propria	25	NA

NA: not available.

**Table 4:** Histopathological immunohistochemistry results and prognosis of patients with intestinal schwannoma after surgery.

Cases	S-100	CD117	DOG-1	CD34	Ki67	Desmin	SMA	Complications	Days of hospitalization	Recurrence or metastasis
1	(+)	(-)	(-)	(-)	(<1%+)	(-)	(-)	No	17	No
2	(+)	(-)	(-)	(-)	(3%+)	(-)	(-)	No	15	No
3	(+)	(-)	(-)	(-)	(1%+)	(-)	NA	Obstruction	17	No
4	(+)	(-)	(-)	(-)	(3%+)	(-)	(-)	No	12	No
5	(+)	(-)	(-)	(-)	(1%+)	(-)	(-)	No	12	No
6	(+)	(-)	(-)	(+)	(<1%+)	(-)	(-)	No	7	No
7	(+)	(-)	(-)	(-)	(1%+)	(-)	(-)	Ascites	26	No
8	(+)	(-)	(-)	(-)	(<1%+)	(-)	(-)	No	15	No
9	(+)	(-)	(-)	(-)	(2%+)	(-)	(-)	No	13	No

NA: not available.

## Data Availability

The clinicopathological data used to support the findings of this study are included within the article.

## Abbreviations

GIST: Gastrointestinal stromal tumor
EUS: Endoscopic ultrasonography
CT: Computed tomography
CEA: Carcinoembryonic antigen
CA 19-9: Carbohydrate antigen 19-9
NSE: Neuron-specific enolase
SMA: Smooth muscle actin
HPF: High-power fields
WHO: World Health Organization
GIMTs: Gastrointestinal mesenchymal tumors
GANTs: Gastrointestinal autonomic neurogenic tumors
MRI: Magnetic resonance imaging
EUS-FNA: Endoscopic ultrasonography-guided fine needle aspiration or biopsy
PET-CT: Positron emission tomography/computed tomography
GFAP: Glial fibrillary acidic protein
NCCN: National Comprehensive Cancer Network.
